# Genetics of behavioural evolution in giant mice from Gough Island

**DOI:** 10.1098/rspb.2022.2603

**Published:** 2023-05-10

**Authors:** Jered A. Stratton, Mark J. Nolte, Bret A. Payseur

**Affiliations:** Laboratory of Genetics, University of Wisconsin-Madison, Madison, WI 53706, USA

**Keywords:** island syndrome, house mice, anxiety, island evolution, island tameness, behaviour

## Abstract

The evolution of behaviour on islands is a pervasive phenomenon that contributed to Darwin's theory of natural selection. Island populations frequently show increased boldness and exploration compared with their mainland counterparts. Despite the generality of this pattern, the genetic basis of island-associated behaviours remains a mystery. To address this gap in knowledge, we genetically dissected behaviour in 613 F2s generated by crossing inbred mouse strains from Gough Island (where they live without predators or human commensals) and a mainland conspecific. We used open field and light/dark box tests to measure seven behaviours related to boldness and exploration in juveniles and adults. Across all assays, we identified a total of 41 quantitative trait loci (QTL) influencing boldness and exploration. QTL have moderate effects and are often unique to specific behaviours or ages. Function-valued trait mapping revealed changes in estimated effects of QTL during assays, providing a rare dynamic window into the genetics of behaviour often missed by standard approaches. The genomic locations of QTL are distinct from those found in laboratory strains of mice, indicating different genetic paths to the evolution of similar behaviours. We combine our mapping results with extensive phenotypic and genetic information available for laboratory mice to nominate candidate genes for the evolution of behaviour on islands.

## 1. Introduction

Organisms on islands behave differently from their mainland counterparts [1–3]. Island environments present novel resources, predation pressures and competition [4,5], leading to the evolution of new foraging strategies and activity patterns in island colonizers [6,7]. Investigating the genetic basis of behavioural evolution in island populations has potential to reveal mechanisms by which organisms respond to environmental change.

Behaviour of insular populations has long been of interest to evolutionary biologists. Darwin first used the term ‘island tameness’ [1] to describe the reduction in anti-predator behaviours commonly shown in insular organisms [2,3,8]. As part of the ‘island syndrome’ [9], insular populations show reduced aggression compared with mainland conspecifics [10–12]. The ecological conditions that drive novel behaviours in insular populations, such as island area, island remoteness and predator prevalence, have been subjects of many investigations [13–17]. However, few studies have shown a heritable component to island-associated behaviours [18,19], a requirement for evolution and the identification of causative loci.

A striking example of an insular population with documented, heritable changes in behaviour is the house mice that invaded Gough Island (hereafter ‘GI’) [20], a remote island in the middle of the South Atlantic. These mice belong to the subspecies *Mus musculus domesticus* [21] and colonized the remote island probably via sailing vessels from Western Europe [22,23] a few hundred to a few thousand generations ago [24,25]. Since colonization of GI, the mice have evolved an exceptionally large size [26,27], a pervasive expansion of the skeleton [28], a stronger bite [29] and a new mandible shape [30]. These unusual phenotypes echo the unusual ecology of the island, where the mice live without predators or human commensals [24] and feed primarily on invertebrates and seeds [26]. In the winter, GI mice predate on seabird populations, consuming an estimated 2 million chicks and/or eggs per year [31]. Following expectations for island colonizers and corresponding to their novel predatory behaviour, GI mice are bolder and more exploratory in novel environments compared with mainland conspecifics [20]. A genetic investigation of these behavioural changes promises to provide insight into long-standing questions about behavioural evolution.

In addition to being important parameters for fitness, behaviours associated with island adaptation, including reduced anxiety, are related to those underlying human behavioural disorders [32,33]. Classical inbred strains of house mice are established models for human behaviour [34] and are routinely used to study its genetic architecture [35–37]. Cross-species comparisons have successfully identified candidate genes underlying behaviour in both mice and humans [38–40]. Despite this wealth of knowledge from laboratory strains of house mice and the expectation that natural populations will provide new insights into the inheritance of complex traits [41], genetic examinations of behavioural evolution in wild mice remain rare. In this study, we use GI mice as a model system to characterize the genetic architecture of the evolution of boldness and exploration on islands.

## 2. Material and methods

### (a) Mouse strains and husbandry

**Figure 1.**
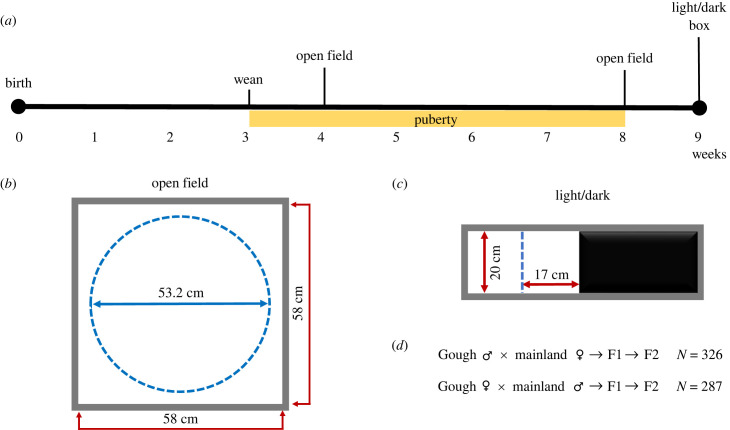
Summary of behavioural phenotyping timeline and assays. (*a*) Timeline of a subject mouse's life. (*b*) Schematic of the open field arena. The centre defined during video analysis is outlined by the dashed circle. (*c*) Schematic of the light/dark box. The subject had free access to both equally sized chambers during the test. Distance to the centre threshold is noted by the dashed line. (*d*) Cross design and sample sizes. F1s were generated in both maternal cross directions. F2s were generated through sibling matings of F1s.

To generate F2 mice, we conducted intercrosses between inbred strains of GI mice (produced in our laboratory) and mainland mice, the same strains used by Stratton *et al*. [20]. In 2009, GI mice were live-caught and shipped to the University of Wisconsin School of Veterinary Medicine Charmany Instructional Facility, where a breeding colony was established [27]. GI mice were then bred for over 20 generations of brother–sister mating to establish an inbred line. Mainland mice belong to the WSB/EiJ inbred strain (JAX stock no. 001145), founded from breeding pairs caught in Maryland, and were maintained in the same colony as GI mice for the same period of time. GI mice and house mice from the eastern coast of North America are likely descended from Western European populations [21,42], though the geographical locations of specific source populations are unknown. Husbandry and weighing protocols were identical to those applied by Stratton *et al*. [20] (see electronic supplementary material, file S1). F1s were generated by crossing mice from the GI strain and the mainland strain in both maternal directions (figure 1*d*). F1 siblings were crossed to each other to generate F2s.

### (b) Behavioural assays

All behavioural assays were conducted in a similar manner to Stratton *et al*. [20], restated in electronic supplementary material, file S1 with differences noted. Phenotyping was conducted from January 2020 to July 2021. Each subject was tested three times with the following regimen (figure 1*a*): open field (4 and 8 weeks old ±1 day; figure 1*b*) and light/dark box (9 weeks old ±1 day; figure 1*c*). Videos were recorded for each test (for 30 min) and run through a modified ImageJ script associated with MouseMove [43] to create trajectory files with *x* and *y* coordinates of the mouse at each frame. To conduct function-valued trait analyses, behavioural measurements were extracted from trajectory files at every minute of the test using an independent pipeline (see electronic supplementary material, file S1). After each assay, the number of faecal boli in the arena was counted ('faecal boli'). For ‘time in centre’, the centre of the open field was defined as a circle with a 26.59 cm radius (14.5 cm radius in Stratton *et al.* [20]) that stretches to 1 inch from the edge of the arena. ‘distance travelled’ was the sum of all positional changes above the movement threshold determined by the method of Shoji [44]. All computational scripts used in video analysis are available in electronic supplementary material, files S3-S9.

Time spent past two different thresholds in the light chamber were recorded: (i) 1.27 cm (0.5 inch) from the dark chamber, ('time in light chamber') and (ii) midpoint of the light chamber ('time past centre'). The number of crosses past these thresholds was also recorded (‘light chamber entrances’ and ‘centre crosses’ respectively). Raw measurements and associated metadata for each F2 are included in electronic supplementary material, table S2.

### (c) Genotyping

Liver tissue was collected from each F2 and submitted to Neogen for genotyping on the Giga Mouse Universal Genotyping Array (https://www.neogen.com/categories/genotyping-arrays/gigamuga/, see electronic supplementary material, file S1). Previous versions of this array were used successfully to map quantitative trait loci (QTL) for morphological traits between GI mice and WSB/EiJ mice [27,28,30]. After quality control checks (see electronic supplementary material, file S1), a final dataset of 613 F2s with genotype information at 31 681 informative markers was used for QTL analyses.

### (d) Quantitative trait loci analyses

All statistical analyses were conducted in R (v 3.4.1; v 4.1.3 for function-valued trait analyses and scans for epistasis) [45]. Two approaches were taken to identify QTL for behaviour (see electronic supplementary material, file S1): (i) single-trait (hereafter ‘ST’) analyses using the value of each phenotype at the end of the behavioural assay, and (ii) function-valued trait analyses combining information across the entire behavioural assay.

### (e) Single-trait mapping

ST analyses were conducted in R/qtl [46] using the value of each behavioural trait from the culmination of the assay. Analyses used Haley–Knott regression [47] with covariates listed in the electronic supplementary material, table S1. To determine which covariates to include in the QTL scan, environmental and pedigree-related predictors were evaluated using linear models for each trait. Independent variables were included as additive covariates when deemed significant by an additional sum-of-squares test comparing models that included or excluded the variable. To search for QTL-by-sex interactions, we conducted scans with sex as an interactive covariate for traits where sex was included as an additive covariate (time in centre juvenile, distance travelled juvenile, distance travelled adult, faecal boli adult, light chamber entrances, centre crosses and faecal boli total).

### (f) Function-valued trait mapping

To incorporate temporal dynamics of behaviours during the course of the assay, we conducted function-valued trait QTL mapping as implemented in r.funqtl [48]. Faecal boli were counted only at the end of each assay and were not included in this analysis. Values for each behaviour were calculated at each minute of the test (1–30) and the collection of these values was converted to a set of functional principal components (fPCs). The fPCs were then fitted to the same covariates used in ST analyses and residuals were extracted for QTL mapping. Three different mapping approaches were used. ‘HK’ multivariate mapping follows the method of Knott & Haley [49]. ‘SL’ mapping and ‘ML’ mapping scan each fPC individually and extract the average and maximum logarithm of odds (LOD) scores, respectively. These three approaches are designed to capture QTL with a wide range of effect sizes and durations of activity.

### (g) Scans for epistasis

We applied two methods to search for epistatic QTL (see electronic supplementary material, file S1). First, we conducted scans for pairs of interacting QTL using the function scantwo() in R/qtl [46]. All pairs of autosomal locations were considered. Second, we searched for QTL with differences in variance among genotypes using the function scaneonevar() in R/qtl.

### (h) Candidate gene nomination

Candidate genes for each QTL were nominated based on three primary criteria (see electronic supplementary material, file S1): (i) associations with phenotypes of interest in mice and humans, (ii) sequence differences between the two inbred strains used in the cross, and (iii) expression in brain regions of interest in mice.

### (i) Evidence for selection

To examine evidence for positive, directional selection acting on candidate genes in GI mice, we compared our findings with those presented in Payseur & Jing [25]. These authors computed posterior probabilities of directional selection across the genome using 14 wild-caught GI mice and their mainland counterparts (see electronic supplementary material, file S1). We extracted the maximum posterior probability within each candidate gene region to evaluate evidence for selection acting on behavioural candidate genes.

## 3. Results

### (a) Phenotypic variation

**Figure 2.**
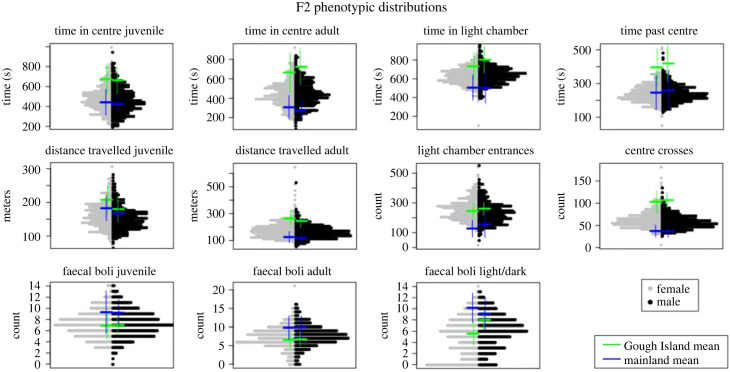
Summary of behavioural distributions. Distribution of F2 phenotypes across three assays. Means (±1 s.d.) of GI mice and means (±1 s.d.) of mainland mice (extracted from data in Stratton *et al.* [20]) are noted by green and blue bars, respectively.

Stratton *et al*. [20] demonstrated that GI mice and mainland mice show extensive behavioural differences across assays (figure 2, green and blue bars). GI mice spend more time in the centre of the open field, deposit fewer faecal boli and explore the light chamber more often than mainland mice. GI mice travel more in the open field than mainland mice, but primarily as adults. All behavioural traits show approximately normal distributions across F2 mice, except for distance travelled as adults (which is right-skewed) (figure 2). Means of F2 distributions are often shifted away from the midparent value (the arithmetic average of parental strain means), with the direction of this shift varying among traits. F2 means resemble GI mice for counts of faecal boli (across all assays) and for the number of light chamber entrances. F2 means and mainland mouse means are more similar for time spent in the centre of the open field and time spent past the centre of the light/dark box.

### (b) Correlations among traits

**Figure 3.**
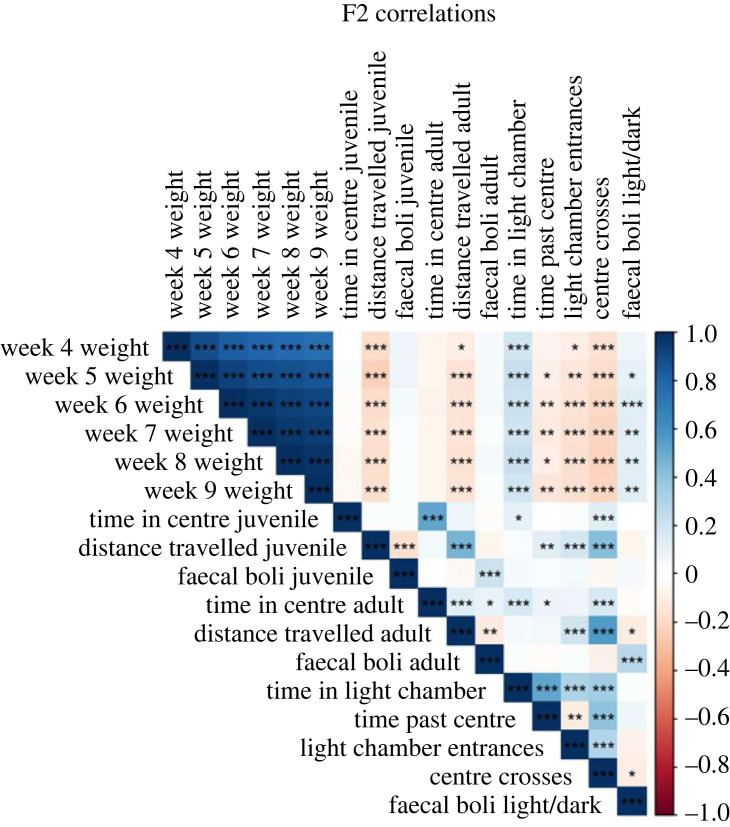
Pairwise correlations among body size and behavioural traits across F2s. Correlation significance is denoted by the number of asterisks: *, *p* < 0.05, **, *p* < 0.01, ***, *p* < 0.001.

To examine relationships among phenotypes, we calculated pairwise correlations between all traits across F2s (figure 3). The same traits measured in juveniles and adults using the open field test are positively correlated. The number of centre crosses in the light/dark box is positively correlated with distance travelled in the open field at both juvenile (*r* = 0.425, Pearson's product–moment correlation; *p* < 0.001) and adult (*r* = 0.568; *p* < 0.001) stages, suggesting these traits capture the same underlying behaviour. Body size is weakly negatively correlated with distance travelled at both ages (juvenile: *r* = −0.182; *p* < 0.001; adult: *r* = −0.151; *p* < 0.001) and with the number of centre crosses in the light/dark box (*r* = −0.219; *p* < 0.001), but size is uncorrelated with faecal boli counts in the open field test (*p* > 0.05). Notably, different behaviours within the open field test have correlations near zero. Faecal boli counts in the light/dark box are also uncorrelated with other behaviours in the light/dark box. Together, these results cluster behaviours into three groups: (i) anxiety, captured by time in the centre of the open field and time in the light chamber of the light/dark box, (ii) exploration, captured by distance travelled in the open field and centre crosses in the light/dark box, and (iii) defaecation, measured by faecal boli counts. Correlations between traits within an assay decrease across timepoints (electronic supplementary material, figure S1). Such declines are expected when the underlying functions for each trait are approximately linear with time and with different slopes. Overall, these findings suggest that the suite of behaviours we quantified are genetically separable and context-dependent.

### (c) Quantitative trait loci for behavioural evolution

**Figure 4.**
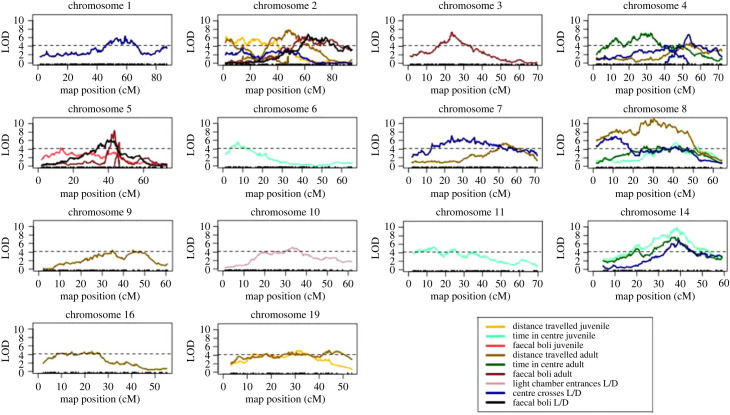
LOD profiles of QTL identified by ST mapping. Only chromosomes where a QTL was identified are shown. The maximum 5% significance threshold among traits (4.19) is shown by a dashed line (see electronic supplementary material, file S1). Thresholds for other traits are similar, with a minimum of 3.93.

We identified a total of 41 QTL across ages, traits and analyses (table 1; figure 4). We uncovered no evidence for epistatic QTL. Our experimental design provides an unusual opportunity to compare the genetics of the same behaviour at two developmental stages. Although common QTL sometimes affect the same trait measured in juveniles and adults, genetic architectures differ at the two ages. For example, whereas more QTL modulate distance travelled and the number of faecal boli in adults, more QTL regulate time in centre in juveniles.

**Table 1.** Summary of QTL for behaviour.

assay	age	trait	chromosome	position (Mb)^a^	lower bound^b^	upper bound^c^	methods detected^d^	Gough parental direction^e^	additive direction^f^	dominance direction^g^
open field	juvenile	time in centre	2	94.11	88.63	99.32	HK, SL	**− : +**	**−**	**−**
			3	72.58	67.68	81.26	HK	**− : +**	**−**	**−**
			6	27.52	16.05	39.46	ST, ML	**− : +**	**+**	**−**
			8	104.58	91.58	119.60	ST	**− : +**	**+**	**+**
			11	34.38	7.12	67.77	ST	**− : +**	**+**	0
			14	90.32	80.55	98.37	ST, HK, ML, SL	**− : +**	**+**	**+**
		distance travelled	1	126.2	113.44	137.15	HK	**+**	**+**	**+**
			2	5.99	3.18	69.75	ST, HK, ML, SL	**+**	**+**	0
			14	62.13	14.61	79.21	HK	**+**	**−**	0
			19	43.32	20.36	49.72	ST, ML, SL	**+**	**−**	0
		faecal boli	5	30.63	21.77	108.62	ST	**−**	**−**	**−**
	adult	time in centre	4	84.68	35.93	91.39	ST, ML, SL	**+**	**+**	**−**
			8	109.4	45.29	118.22	ST, HK	**+**	**+**	0
			14	86.92	76.4	93.42	ST, HK, ML, SL	**+**	**+**	**+**
		distance travelled	2	11.94	3.18	27.73	ST	**+**	**+**	0
			2	99.01	77.16	123.60	ST, HK, ML, SL	**+**	**+**	0
			4	57.65	37.55	65.27	SL	**+**	**−**	0
			4	133.5	123.81	139.01	ST, HK, ML, SL	**+**	**+**	0
			7	129.45	120.16	137.37	ST, ML, SL	**+**	**−**	0
			8	78.96	60.36	100.12	ST, HK, ML, SL	**+**	**+**	**−**
			9	105.28	69.38	115.44	ST, HK, ML, SL	**+**	**+ : −**	0
			14	99.33	75.05	119.69	ML	**+**	**+**	0
			16	45.59	9.07	57.56	ST, HK, ML, SL	**+**	**+**	0
			19	57.99	14.58	60.23	ST, ML, SL	**+**	**−**	**−**
		faecal boli	2	130.24	113.07	170.75	ST	**−**	**−**	**−**
			3	74.11	71.97	81.94	ST	**−**	**+**	**−**
			5	106.31	103.17	107.39	ST	**−**	**−**	**−**
			5	113.5	110.66	114.64	ST	**−**	**+**	**+**
light/dark box		time in light chamber	5	112.86	110.54	130.70	HK	**+**	**+**	**+**
			8	33.7	26	42.02	HK, SL	**+**	**+**	**+**
		time past centre	9	107.37	91.3	121.17	HK	**+**	**− : +**	**−**
		light chamber entrances	10	91.48	54.07	99.46	ST, ML	**+**	**−**	0
		centre crosses	1	157.98	126.96	167.82	ST (females)	**+**	**+**	0
			2	27.1	3.18	124.85	ST, HK	**+**	**+**	**+**
			4	102.71	57.76	120.17	ST	**+**	**−**	**−**
			4	132.64	129.6	134.31	ST	**+**	**+**	**+**
			7	63.32	54.2	108.92	ST, HK, ML, SL	**+**	**−**	**−**
			8	31.08	10.79	36.30	ST, HK, ML, SL	**+**	**+**	0
			14	93.07	79.92	98.37	ST, HK, SL	**+**	**+**	**+**
		faecal boli	2	137.4	133.02	164.48	ST	**−**	**−**	0
			5	98.66	80.13	108.86	ST	**−**	**−**	**−**

^a^Position of QTL in megabases (Mb) based on the GRCm39 build of the house mouse genome. ^b^Position of lower bound in megabases from 1.5 LOD confidence interval. ^c^Position of upper bound in megabases from 1.5 LOD confidence interval. ^d^Methods that identified the QTL: ST = single-trait analysis, HK = multivariate function-valued trait analysis, SL = average LOD score of independent QTL scans from function-valued trait analysis, ML = maximum LOD score of independent QTL scans from function-valued trait analysis. ^e^Sign of GI mouse strain mean minus mainland mouse strain mean. Colon indicates change from early to late timepoints of the assay. ^f^Sign of additive effect of substituting a GI mouse allele. Colon indicates change from early to late timepoints of the assay. The 95% confidence interval of the effect must not overlap zero for a change in sign to be reported. ^g^Sign of dominance effect (mean of heterozygotes minus the midpoint between the means of the two homozygote classes). If the 95% confidence interval for the effect overlapped zero a ‘0’ is reported.

Different traits measured at the same age also show distinct genetic architectures (table 1). In juveniles, time in centre, distance travelled and number of faecal boli have no QTL in common. Although QTL confidence intervals on chromosomes 2, 4, 8 and 14 overlap for open field traits quantified in adults, the best-estimated positions of these QTL are far apart and confidence intervals are challenging to estimate, especially for methods that incorporate information across timepoints during the experiment. The QTL on chromosome 19 is unique to distance travelled in the open field. The QTL on chromosome 7 is unique to exploration in adults. Most QTL are distinct across behavioural traits measured within the juvenile open field test and light/dark box test. The QTL on chromosome 1 is associated with both sexes in juveniles but with females in adults for the light/dark box. Our application of four statistical approaches to QTL mapping (see ‘Single-trait mapping’ (§2e) and ‘Function-valued trait mapping’ (§2f) in Methods) expanded the variety of QTL we discovered (table 1). Although many QTL were identified by all four methods, each method detected at least one unique QTL and some QTL were supported by two or three methods. Variation among QTL across methods likely reflects differences in the ability to capture the underlying behaviour. Function-valued trait mapping and ST mapping operated on different data for the same trait, with the former incorporating measurements from every minute of an assay and the latter restricted to cumulative, endpoint measurements. Overall, most QTL appear to be context-specific, detected only at specific ages, in certain assays, or by individual methods.

### (d) Additive effects of quantitative trait loci

**Figure 5.**
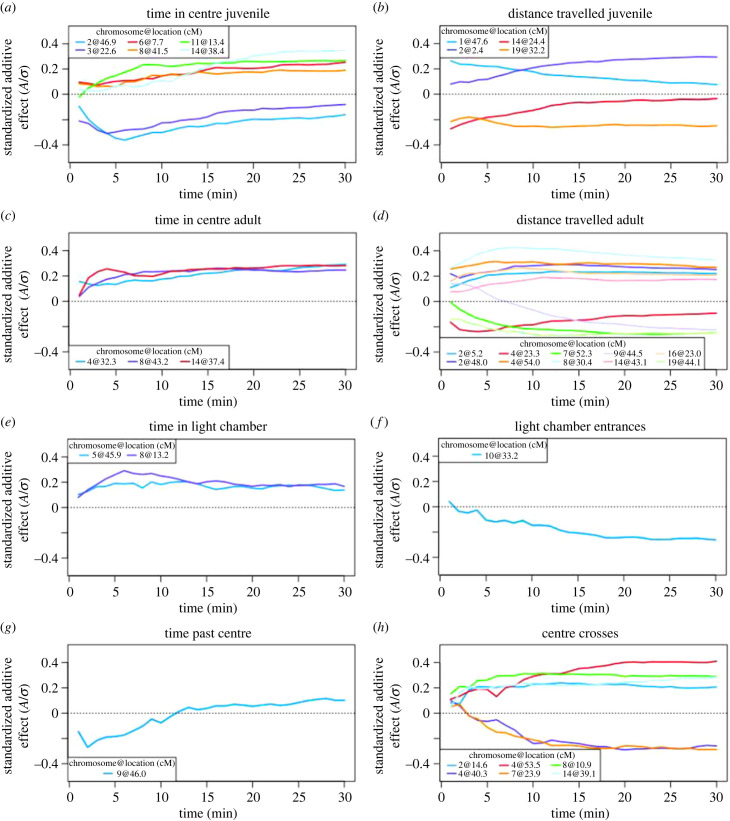
Standardized additive effects of QTL. Additive effects of QTL were jointly estimated at every minute of the assay (1–30). Each effect was standardized by the phenotypic s.d. (*a*) Time spent in the centre of the open field as juveniles. (*b*) Distance travelled in the open field as juveniles. (*c*) Time spent in the centre of the open field as adults. (*d*) Distance travelled in the open field as adults. (*e*) Time spent in the light chamber of the light/dark box. (*f*) Number of entrances to the light chamber. (*g*) Time spent past the centre of the light/dark box. (*h*) Number of crosses past the centre of the light/dark box.

**Figure 6.**
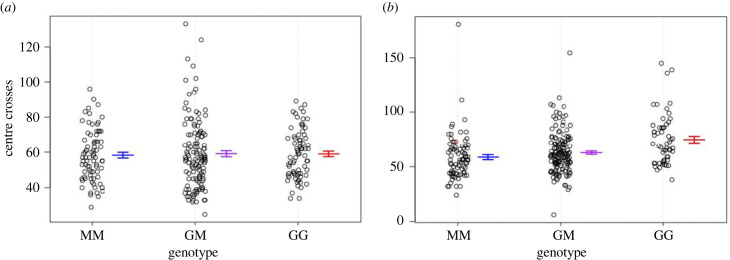
Effect plot of female-specific QTL on chromosome 1. The number of centre crosses in the light/dark box for males (*a*) and females (*b*) within each genotypic class is plotted for the marker at the peak of the QTL. Bars denote means of genotypic classes ±1 s.e. ‘MM’ = homozygous mainland alleles, ‘GM’ = heterozygous, ‘GG’ = homozygous GI alleles.

One characteristic shared across QTL is their moderate effect size. Additive effects are typically less than 0.4 phenotypic s.d. (figure 5; table 2). However, these effects are associated with noticeable shifts in observed behaviours (electronic supplementary material, figure S2). Strikingly, the magnitude of the additive effect of a QTL often changes across timepoints within an assay, with some QTL showing peak effects at early timepoints and others increasing in magnitude over time (figure 5*a,b*). GI mouse alleles at the QTL on chromosome 3 in juveniles decrease the amount of time spent in the open field by approximately 10 s after 15 min of exploration, but do not cause any additional effects for the remainder of the assay (electronic supplementary material, figure S2A). By contrast, time spent in the light chamber increases by approximately 0.5 s per min for each GI mouse allele at the two QTL on chromosomes 5 and 8 (electronic supplementary material, figure S2E). Whether the GI mouse allele increases or decreases the trait value varies across QTL (figure 5; electronic supplementary material, table S3 and figure S2); the direction of additive effects and the direction of the difference in parental means do not consistently match (table 1). Additive effects at most QTL maintain their sign across the entire assay, including those for time in centre as juveniles, a trait for which the difference in parental means changes during the assay (table 1; electronic supplementary material, figure S3A). The additive effect of the QTL on chromosome 9 shifts in sign during the assay, though the pattern is reversed between distance travelled in the open field and time past centre in the light/dark box (table 1; figure 5*d*,*g*). The effect of the QTL on chromosome 1 for centre crosses in the light/dark box is restricted to females (figure 6).

**Table 2.** Standardized additive effects for faecal boli QTL. Sign of effect reflects the action of a GI mouse allele. Note that the GI strain deposited fewer faecal boli than the mainland strain in all assays (figure 2).

assay	chromosome	location (cM)	standardized additive effect (*A*/*σ*)^a^
open field juvenile	5	13.06	−0.221
open field adult	2	58.161	−0.244
	3	23.363	0.309
	5	43.491	−0.604
	5	46.257	0.379
light/dark box	2	62.55	−0.303
	5	39.586	−0.219

^a^Additive effect of QTL divided by the s.d.

### (e) Dominance effects of quantitative trait loci

Dominance is present across all traits, with effects in both directions (table 1). Dominance is less apparent for QTL associated with distance travelled in the open field (table 1). Among QTL for anxiety-related traits (time in centre, time in light chamber and faecal boli) with consistent signs of effects across the assay and evidence of dominance, GI mouse alleles are dominant more often than expected by chance (12 out of 15 QTL; *p* = 0.035; two-tailed cumulative binomial probability). We present qualitative results in table 1 as dominance effects vary in magnitude across traits and timepoints with substantial s.e. (electronic supplementary material, file S2).

### (f) Contributions to strain differences

Collectively, the additive effects of QTL identified for each trait explain, on average, 44.1% of the difference in means between GI mice and mainland mice at the end of each assay (electronic supplementary material, file S2 and figure S4). The per cent difference explained varies substantially among traits (electronic supplementary material, file S2 and table S3). In juveniles, QTL for time spent in the centre of the open field explain 93.7% of the difference in means between GI mice and mainland mice at the final timepoint, whereas the single QTL for time spent past the centre of the light chamber explains only 7.4% of the parental strain difference.

### (g) Candidate genes

We identified strong candidate genes for each QTL (electronic supplementary material, table S4). Two thousand and ninety-eight genes (electronic supplementary material, table S5) were pulled down in the initial filtering step for associations to behaviour (see electronic supplementary material, file S1). Of these 2098 genes, electronic supplementary material, table S4 highlights 24 genes with relevant sequence differences between GI mice and mainland mice (i.e. protein-coding changes or changes in regions conserved across placental mammals). These genes were chosen based on three criteria: (i) known influences on behaviours of interest in mice and humans, (ii) proximity of the gene to the peak position of the QTL, and (iii) the ubiquity of expression in three brain regions known to be involved in anxiety and exploration (i.e. the hippocampus, amygdala and prefrontal cortex).

### (h) Evidence for natural selection

To explore whether selection contributed to the evolution of behavioural QTL in GI mice, we compared our candidate genes with posterior probabilities of selection calculated in wild populations of GI mice and mainland mice [25]. Three candidate genes overlap 5 kb windows with strong signatures of selection (posterior probability ≥0.9): *Dab1, Cadps2* and *Syn3*. *Lsamp* overlaps a window with a posterior probability of selection of 0.584. None of the sequence differences listed in electronic supplementary material, table S4 falls in these windows with maximum posterior probabilities of selection.

## 4. Discussion

We provide a rare glimpse into the genetics underlying island-associated behavioural evolution. Our study draws on the vast resources and knowledge base of laboratory house mice to improve the growing understanding of behavioural evolution [50–53]. The evolution of striking behavioural differences between GI mice and mainland mice involved multiple genetic changes with individually modest phenotypic effects. The genetic architecture we uncovered emphasizes the importance of context for boldness and exploration, with the action of many loci localizing to certain ages, assays, or timepoints within assays.

Our study highlights advantages of designing experimental assays and statistical analyses to capture dynamic genetic effects underlying transitory behaviours. Extending the period of observation to 30 min, a longer interval of free exploration than is typical in open field and light/dark box experiments, enables the measurement of both initial and acclimatized responses to novel environments. Assessing the same mice at two ages yields a developmental perspective on behaviours whose connection to fitness is expected to differ between juveniles and adults. Function-valued trait mapping considers the trajectory of behaviour across an assay, a rich description that makes it possible to identify loci with phenotypic effects that change over time.

Correlations among phenotypes across F2s provide insights into the process of behavioural adaptation in GI mice. Behaviours separated into three distinct groups: anxiety, exploration and defaecation. The QTL we detected largely echo this grouping, suggesting evolution proceeded independently within each category. Interestingly, body size is negatively correlated with exploration, a pattern also seen by Zhang & Gershenfeld [54]. Increased body size and enhanced exploration are expected to be associated with higher fitness in GI mice. In this scenario, the negative correlation between these traits may have created conflict during their evolution. This inference is consistent with the ‘constraint’ hypothesis for the evolution of syndromes described and demonstrated in field crickets [55]. However, exploration may not be an important determinant of fitness on the island if resources are readily available, allowing body size to evolve more rapidly.

We expand the extensive genetic dissection of house mouse behaviour into wild populations. Mapping studies using classical inbred strains of house mice revealed QTL influencing behaviour scattered across the genome [37]. Notably, chromosomes 1, 15 and 18, which show dense clusters of QTL influencing emotionality in the open field test [56], are underrepresented in our results. This discordance suggests that natural populations and classical inbred strains of mice followed distinct genetic paths to evolve similar behaviours, and highlights the potential of wild-derived strains to identify novel candidate genes influencing anxiety and exploration. Still, some of our findings resemble those for laboratory mice. In one example, Turri *et al.* [57] measured 22 phenotypes across nine behavioural assays in over 1600 F2s created by intercrossing the DeFries mouse strain selected for high activity in the open field with the DeFries strain selected for low activity [58]. Resembling our results, Turri *et al.* [57] found many QTL that are context-specific, with a few QTL that affect multiple behaviours. Furthermore, these authors noted that the QTL with the largest effect on anxiety had no effect on defaecation, echoing our findings that suggest these traits are genetically separable. Faecal boli counts might capture other forms of anxiety (called ‘emotional elimination’ by Turri *et al.* [57]) or be more closely connected to metabolism.

We used multiple lines of evidence to identify strong candidate genes for the evolution of behaviour. Several candidate genes are involved in evolutionarily ancient signalling processes in the brain, including the glutamate receptors *Gria2* and *Grm2* [59]. Other candidate genes, such as *Dab1*, *Eya3*, *Mapk10*, *Lsamp*, *Cadps2* and *Slit1*, function in neuronal development and/or axon guidance [60–65]. These two categories of genes, those expressed at the time the behaviour is enacted and those involved in development at earlier stages, underscore the question of which biological processes are altered when boldness and/or exploration evolve in nature.

Our findings also shed light on how evolution shapes behaviour. The direction of additive effects (i.e. whether the GI mouse allele increases or decreases the trait) varies across QTL. The mixture of QTL effects contributes to the transgressive distribution of behaviours in F2s, where phenotypic ranges exceed the parental means (figure 2). This pattern argues against directional selection toward a consistent optimum as a primary cause of the full suite of behaviours we examined [66]. Nevertheless, additive effects of QTL for anxiety-related traits (i.e. time in centre of the open field, time in the light chamber) all align with parental differences. This finding supports the proposal that bolder mice are more fit on GI because they spend less time and energy on costly predator avoidance behaviour in an environment without predators [7]. Additionally, increased boldness may enhance novel predation of seabird chicks, an important source of food during the winter [31,67]. Evolution of these traits may have been accentuated by dominance favouring GI mouse alleles for boldness.

The evolutionary histories of the QTL we identified merit deeper investigation. The genetic changes responsible for QTL could have evolved along either the GI mouse lineage or the mainland mouse lineage. Although the mainland strain we used has been housed in a laboratory environment for several decades, mice from this strain maintain behaviours typical of wild house mice [68]. Comparisons with 62 mouse strains in the Mouse Phenome Database [69] place GI mice in the phenotypic extremes for open field behaviours. As a result, the strains of mice we used should provide good comparisons for revealing the genetic basis of island-associated behaviours.

Our results lay the groundwork for additional questions that future studies may address. First, as is typical for genetic mapping studies that rely on a single generation of recombination, many QTL we identified have broad confidence intervals and may contain multiple causative mutations. Fine-mapping has revealed some behavioural QTL in laboratory mice to be caused by several closely linked genetic changes [56,70]. Development and refinement of congenic strains that harbour GI mouse alleles may reveal additional loci with varying effects. Additionally, the relationships between the behaviours we measured and others associated with the island syndrome are unknown. Reduced aggression is commonly associated with island life in rodents [9,12]. Though we have not measured aggression directly in GI mice, we suspect from rates of injuries in our colony that it is reduced. Since boldness and aggression are thought to be related [71], it would be useful to assess the relationship between these traits in GI mice. Lastly, our inbred strain of GI mice is only a partial representation of the genetic diversity of GI mice. Mice from highland and lowland sites on GI are known to differ in both body size and rates of predation on seabird chicks [67]. Additional investigations among genetically distinct GI mice may reveal new insights into the evolution of behaviour on islands.

Our study provides an initial genetic portrait of the evolution of boldness and exploration on an island with key environmental differences from the mainland. We hope future studies that dissect the genetic causes of island tameness in other populations will enable comparisons among loci underlying this pervasive phenomenon. Identifying genes and variants responsible for behavioural variation begins with pioneering mapping studies and is a vital part of understanding the evolution of behaviour in nature.

## Data Availability

All computational scripts used in video analysis are available in electronic supplementary material, files S3–S9. Raw measurements and associated metadata for each F2 used in QTL analyses are included in electronic supplementary material, table S2. The data are provided in the electronic supplementary material [72].
